# Oncological Outcomes after Elective and Emergency Resection of Small Intestinal Neuroendocrine Tumours

**DOI:** 10.1007/s12029-026-01419-9

**Published:** 2026-02-19

**Authors:** Ibrahim Alibrahim, Denna Fryer, Sameesh Gupta, Anna Peace, Minh Tu Vo, Ankit Jain, Desmond Yip, Sivakumar Gananadha

**Affiliations:** 1 Department of General Surgery, Canberra Hospital, Yamba Drive, Garran, ACT 2605 Australia; 2 Department of Medical Oncology Canberra Health Services, Canberra, Australia; 3 School of Medicine and Psychology, Australian National University, Canberra, Australia

**Keywords:** Small intestinal neuroendocrine tumours (siNET), Midgut neuroendocrine tumours, Emergency surgery, Elective surgery, Oncological outcomes, Carcinoid syndrome

## Abstract

**Purpose:**

Small intestinal neuroendocrine tumour (siNET) has distinct features and disease course compared to other gastrointestinal neuroendocrine tumours. While they mostly present with subtle symptoms, they also can present acutely requiring emergency surgical intervention. We assessed the impact of surgical intervention timing on oncological outcomes of siNET.

**Method:**

A retrospective observational single centre cohort study of all patients diagnosed with siNET, and received surgical intervention at a tertiary level hospital between 2008 and 2025.

**Results:**

A total of 66 patients were diagnosed with SiNET. 22 patients were excluded due to incomplete data (9 patients) and not meeting the eligibility criteria (13 patients). Total of 44 patients met the inclusion criteria, with 30% underwent emergency resection (*n* = 13) and 70% underwent elective resection (*n* = 31). Small bowel obstruction represented the most common cause for emergency presentations (61%), while incidental radiological findings (39%) and carcinoid syndrome (35%) accounted for most elective presentations. Emergency cases were associated with the absence of pre-operative somatostatin receptor imaging, less findings of mesenteric mass (31% vs. 81%), more likelihood of post operative macroscopic residual disease (46% vs. 23%) and disease specific mortality (75% vs. 23%). However, there was no statistically significant difference between the two groups in primary and secondary oncological outcomes. There were no operative or in-hospital mortality in either group as well as no significant difference in complications rates between the groups.

**Conclusions:**

Although emergency surgery was associated with limited preoperative staging and higher proportion of R2 resections, no statistically significant differences in overall survival, recurrence-free survival, carcinoid symptom resolution or local complications were observed. However, these results need to be interpreted cautiously due to the small sample size of the study.

## Introduction

Neuroendocrine tumours (NETs) are heterogenous malignant transformation of the specialised neuroendocrine cells, which can be epithelial or neuroectoderm in origin [1]. Small intestinal NET (siNET) within the jejunum and ileum is the most common subtype of epithelial NETs within gastrointestinal system, and account for 18% of all NET cases [2, 3]. These tumours are biologically and clinically distinct from NETs of other midgut locations (e.g. appendix and large bowel), both in their natural history and management challenges [4, 5].

Small intestinal neuroendocrine tumours are typically slow-growing, but possess a high metastatic potential with 76% of patients presenting with a metastatic disease at initial diagnosis regardless of primary tumour size [3, 6]. Metastasis most frequently involve regional mesenteric lymph nodes and the liver [6]. Non-functioning tumour often present with no symptoms or subtle non-specific abdominal pain, while functioning tumour present with carcinoid syndrome [2]. However, they sometimes present with acute complications such as bowel obstruction, mesenteric ischemia, bowel perforation or gastrointestinal bleeding [7, 8].

The management of siNETs depends on its location, resectability, histological grade, presence of carcinoid syndrome, extent of metastasis and patient functional status [9]. Surgical resection remains the cornerstone of curative treatment with additional goals of controlling local complications (e.g. bowel obstruction and bowel ischemia), carcinoid syndrome and metastatic progression [10]. Current guidelines do not recommend neoadjuvant systemic therapy in resectable localised siNET or adjuvant systemic therapy in resected localised tumours. Systemic therapy is indicated in non resectable locally advanced tumours, metastatic siNET or following an R2 resection. Systemic therapy is utilised for symptomatic management and slowing disease progression either in the form of palliative therapy or adjuvant therapy [2, 9].

Elective resection is generally preferred to allow comprehensive preoperative assessment, multidisciplinary planning, and optimal oncological clearance including adequate lymphadenectomy [11, 12]. However, emergency resections are sometimes unavoidable due to acute complications. There is ongoing debate regarding the impact of emergency resection on long-term oncological outcomes, but few studies assessing siNET solely [8]. Many published studies combine data from NETs of various midgut sites, introducing significant heterogeneity and potentially masking important site-specific differences in prognosis and treatment responses [12].

This study aims to address the unique clinical and pathological features of these tumours and to provide clarity on the oncological outcomes associated with elective versus emergency surgical resection. By excluding NETs of the appendix and large bowel, we aim to minimize confounding and generate data that are directly relevant to the management of jejunal and ileal NETs—a subgroup for which high-quality evidence remains scarce.

## Methods

A retrospective observational cohort study of all patients who underwent surgical intervention for small intestinal neuroendocrine tumours (siNETs) at the Canberra Health services between January 2008 and February 2025. Eligible patients were identified through hospital medical records as well as a prospectively maintained cancer database.

### Data Collection

A comprehensive review of each patient’s clinical records was performed, which included medical imaging, laboratory results, clinic correspondence, discharge summaries, operative records, anaesthetic records, histopathology reports and systemic therapy prescriptions. The study population included adults aged 18 years old or older who underwent surgical resection of primary neuroendocrine tumours within jejunum and/or ileum. Exclusion criteria included patients who received medical therapy only, palliative resections, or who had another concurrent primary malignant tumour at the time of siNET diagnosis .

Emergency surgery was defined as an unplanned surgery within the same admission for acute complications such as bowel obstruction, bowel ischemia, bowel perforation or gastrointestinal bleeding. Elective surgery was defined as a planned admission for operative intervention with a curative intent after outpatient workup. Procedures were categorised by surgical approach as either open resection or minimally invasive laparoscopic resection. Primary tumour resection was performed either in the form of segmental small bowel resection. ileocecal resection and right hemi colectomy.

Extracted variables included demographic information (age at diagnosis and sex), history of presenting complaint, date of diagnosis, American Society of Anaesthesiologists (ASA) physical status, date of surgery, type of surgery, histopathology findings, World Health Organization (WHO) grading system, AJCC 8th edition staging system and adjuvant therapy. Operative morbidity was assessed using documented estimated bloods loss and Clavien-Dindo Classification of surgical complications. Postoperative surveillance data were collected from follow-up clinic records and imaging studies performed up to 31 July 2025. Surveillance imaging included computed tomography (CT), magnetic resonance imaging (MRI), and somatostatin receptor imaging (SRI) modalities such as DOTATATE PET/CT scan or Octerotide scan.

### Oncological Outcomes

**Fig. 1:**
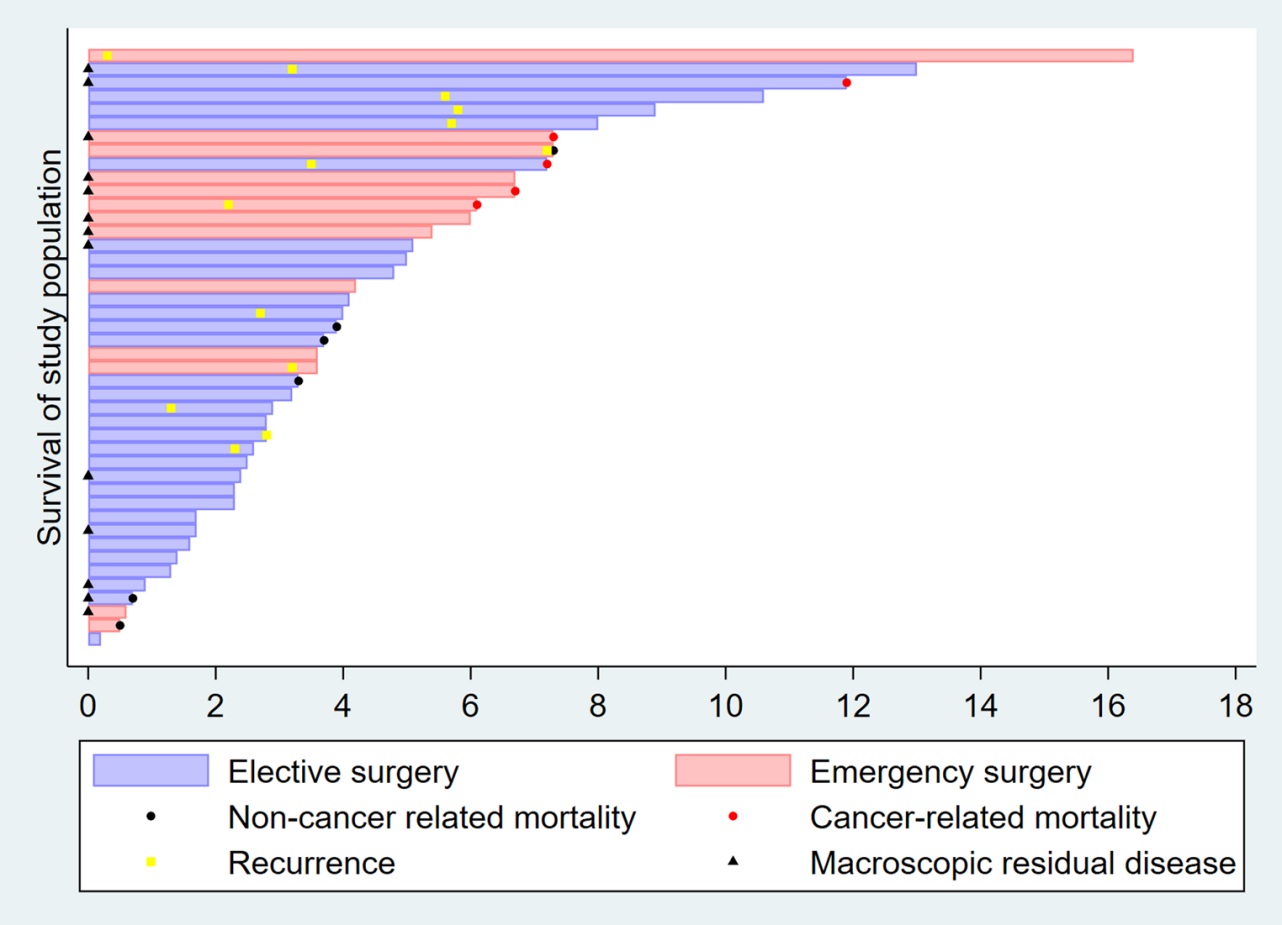
Swimmer plot representing oncological outcomes

Primary oncological outcomes were assessed including residual tumour classification (R status) that was obtained from histopathological report, operative record as well as imaging within 30 days after surgery. R status was defined as R0 (no residual disease), R1 (microscopic residual disease) or R2 (macroscopic residual tumour detected intra-operatively or radiologically). Disease recurrence defined as re-appearance of disease after a documented disease-free interval, confirmed by histopathology or cross-sectional imaging (CT, MRI, or SRI). Further resection was categorised as completion surgery for known residual disease or resection for a recurrent disease. Mortality was categorised as siNET-related or non–siNET-related. siNET-related mortality included deaths due to metastatic spread, tumour mass effect, or complications attributable to advanced disease. Those were recorded based on medical records as well as death certificates. Duration of oncological outcomes were calculated from date of surgery and expressed in years in a swimmer plot graph, as shown in Fig. 1.

Secondary post operative clinical outcomes were assessed including systemic therapy categorised as either adjuvant therapy or post disease progression/recurrence based on the documented indication at the time of commencement. Systemic therapy included somatostatin analogues (SSA), targeted therapy (e.g. Everolimus), peptide receptor radionuclide therapy (PRRT) and chemotherapy. Carcinoid syndrome was defined as the presence of ongoing abdominal pain, persistent diarrhoea, facial flushing, new-onset wheeze and new right sided valvular fibrosis. This was categorised into resolved symptoms, persistent symptoms or new development after disease progression. Bowel obstruction was categorised as malignant small bowel obstruction (due to tumour or mesenteric fibrosis) or non-malignant small bowel obstruction (due to adhesions or other benign causes) based on imaging and operative findings. Bowel ischemia was defined as radiologically or intraoperatively confirmed compromise of bowel vascular supply at any point during follow-up.

### Statistical Analysis

All data were analysed using Stata software. Categorical variables were presented as frequencies and percentages. Continuous variables were expressed as mean ± standard deviation (SD). Independent t-test performed for continuous variable comparison, as all data distribution normality was met. Categorical variables were tested using Chi-square test. A *p*-value of < 0.05 was considered statistically significant.

Survival analyses were performed using the Kaplan–Meier method to estimate recurrence-free survival (RFS), overall survival (OS) and cancer specific survival (CSS), with comparisons between treatment groups made using the log-rank test. Event time was defined from the date of initial surgery to the date of recurrence, death, or last follow-up. Patients without an event at the time of analysis were censored. Cox proportional hazards regression was employed to estimate hazard ratios (HR) with 95% confidence intervals (CI) for OS, adjusting for potential confounders.

## Results

### Study Population

**Fig. 2:**
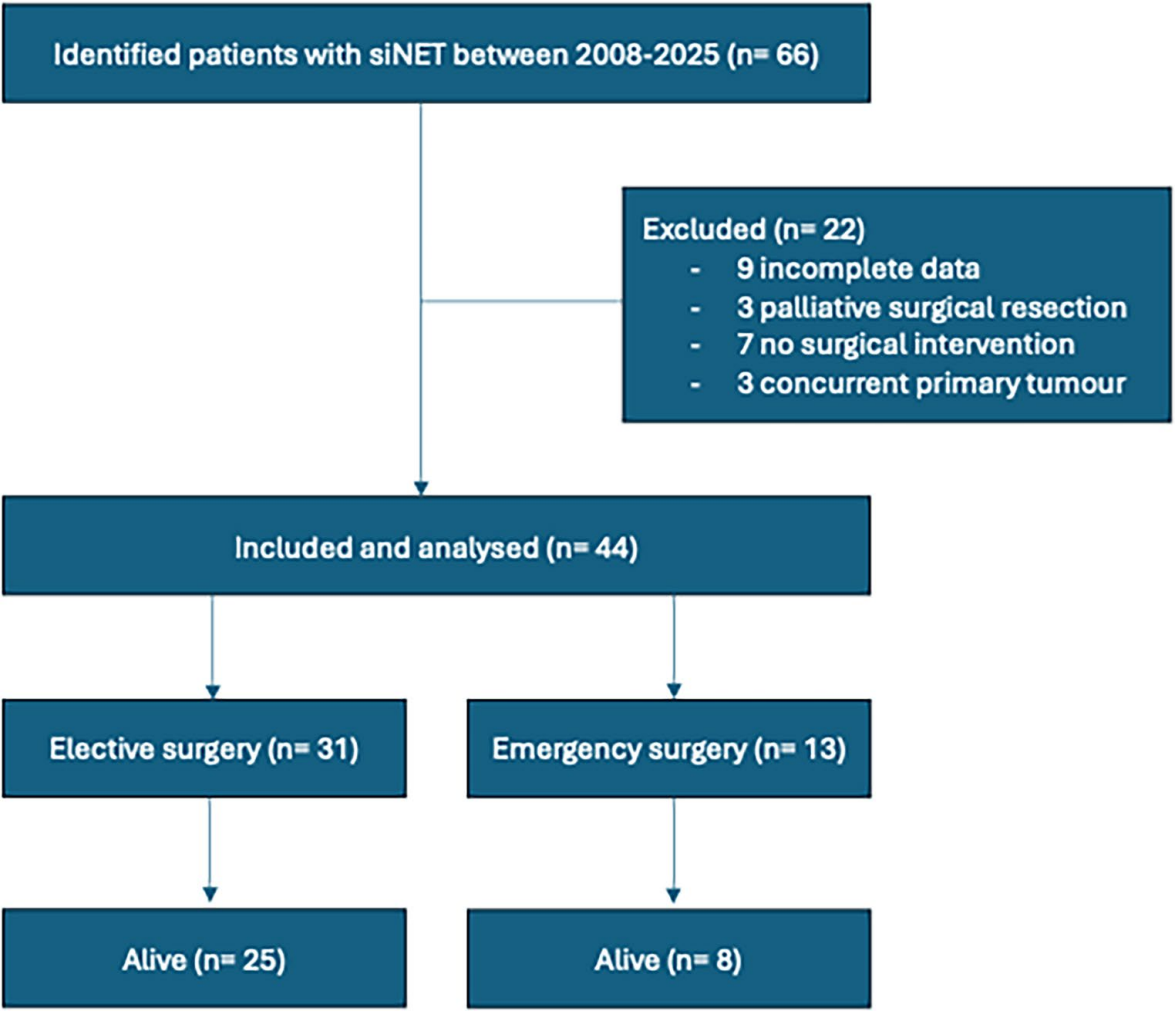
STROBE flow chart

A total of 66 patients were initially identified with siNET between 2008 and 2025, but only 44 patients met the inclusion criteria (Fig. 2). 13 patients (30%) underwent emergency resection and 31 patients (70%) underwent elective resection of a primary siNET.

22 patients were excluded as they did not meet the eligibility criteria with nine patients excluded because no information other than histopathology was available. Thirteen patients were excluded as per criteria with three patients having palliative surgical resection, seven patients receiving only medical management and three paitents having concurrent primary cancer. The latter were assessed for concurrent primary tumour. Two patients had incidental siNET found on histopathology for a colorectal adenocarcinoma. One patient had over two years delayed siNET resection due to concurrent recurrent tongue squamous cell carcinoma and prostatic adenocarcinoma.

### Baseline Characteristics

Baseline features are summarized in Table 1. Patients in the elective group were older than those in the emergency group. There was no statistical difference in either gender distribution or ASA status between the two groups.

Majority of emergency group presented with small bowel obstruction (61%). On the other hand, the elective group presented mostly with incidental radiological findings (39%) or carcinoid syndrome (35%). Pre-operative work up showed more frequent mesenteric mass in elective cases than emergency (81% vs. 31%). As expected, pre-operative somatostatin receptor imaging (e.g. DOTATATE and Octreotide scan) was common in the elective pathway and absent in emergencies (77% vs. 0%). Baseline liver metastases, other sites metastases and carcinoid symptoms at time of presentation were comparable across the two groups.

Elective resections showed higher percentage of multifocal disease compared to the emergency group. Otherwise, histopathological results and surgical morbidity were comparable across the two groups.

**Table 1:** Characteristics of study population

Group	Elective	Emergency	*P*-value*
Count (n | %)	31 | 70%	13 | 30%	
Age at diagnosis, years (mean ± sd)	68 ± 12	56 ± 14	*0.007*
Sex (n | %)			0.851
- Female	11 | 35%	5 | 38%	
- Male	20 | 65%	8 | 62%	
Presentation (n | %)			*0.000*
- Incidental	12 | 39%	0	
- Abdominal pain	8 | 26%	2 | 15%	
- Carcinoid	11 | 35%	0	
- Bowel obstruction	0	8 | 61%	
- Bowel ischemia	0	1 | 8%	
- Gastrointestinal haemorrhage	0	2 | 15%	
Carcinoid syndrome (n | %)	14 | 45%	8 | 62%	0.332
Mesenteric mass (n | %)	25 | 81%	4 | 31%	*0.001*
Liver metastases (n | %)			0.581
- None	22 | 71%	11 | 85%	
- Single	8 | 26%	2 | 15%	
- Multiple	1 | 3%	0	
Other metastases (n | %)	4 | 13%	0	0.174
Pre-operative somatostatin receptor imaging (n | %)	24 | 77%	0	*0.000*
ASA status			0.077
- ASA I	0	1 | 11%	
- ASA II	12 | 44%	2 | 22%	
- ASA III	15 | 56%	5 | 56%	
- ASA IV	0	0	
- ASA V	0	1 | 11%	
Intraoperative Estimated Blood Loss (Median | IQR)	100mL | 100–375mL	100mL | 50-100mL	0.082
Clavien-Dindo Classification			0.76
I	19	8	
II	5	1	
III	5	2	
IV	0	0	
V	0	0	
Unknown	2	2	
Hospital stay (Median | IQR)	7 | 5–13	10 | 8–22	0.12
30 days re-admission	3	0	
Location of primary resected lesion			0.102
- Ileum	12 | 84%	12 | 92%	
- Jejunum	5 | 16%	0	
- Both ileum and jejunum	0	1 | 8%	
Number of resected lesions (% multiple lesions)			*0.029*
- Single	10 | 33%	9 | 69%	
- Multiple	20 | 67%	4 | 31%	
Size of largest resected lesion, mm (mean ± sd)	19 ± 10	24 ± 17	0.207
Grade (n | %)			0.736
- Grade 1	23 | 74%	9 | 69%	
- Grade 2	8 | 26%	4 | 31%	
- Grade 3	0	0	
Resected Margin (n | %)			0.052
- Negative	19 | 63%	12 | 92%	
- Positive	11 | 37%	1 | 8%	
Resected Lymph nodes (n | %)			0.500
- Positive	26 | 96%	10 | 91%	
- Negative	1 | 4%	1 | 9%	
Resected Liver lesions (n | %)			0.062
- No	24 | 77%	13 | 100%	
- Yes	7 | 23%	0	
Resected Mesenteric deposits (n | %)			0.100
- None	11 | 35%	9 | 69%	
- Single	14 | 45%	2 | 15%	
- Multiple	6 | 19%	2 | 15%	

*Independent t-test for continuous variable. Chi-square for categorical variables. Italic indicates significant difference (*P*-value < 0.05)

### Operative Outcomes

One patient in the elective group underwent laparoscopic resection, while the rest of patients among both groups underwent an open resection. There was no stoma creation in any group. There was no significant difference in intra operative blood loss or complication rates between the two groups (Table 1). There was no operative or in-hospital mortality in either group.

### Oncological Outcomes

Primary and secondary oncological outcomes are summarised in Table 2. There was no statistically significant difference between the two groups in regard to overall survival (OS) and recurrence-free survival (RFS), with *p* value of 0.621 and 0.335 respectively.

There was also no statistically significant difference between the two groups in residual tumour status (R status), which was analysed as three separate categories- Elective surgery- R0 14/31 (45%), R1 10/31 (32%), R2 7/31 (23%) and following emergency- R0 6/13 (46%), R1 1/13 (8%), R2 6/13 (46%). Nevertheless, there is a shift from microscopic (R1) to macroscopic (R2) residual in emergency resections that did not reach statistical significance. However, the association between R2 and mortality across the two groups were comparable (2/7 in elective group vs. 2/6 in emergency group).

**Fig. 3:**
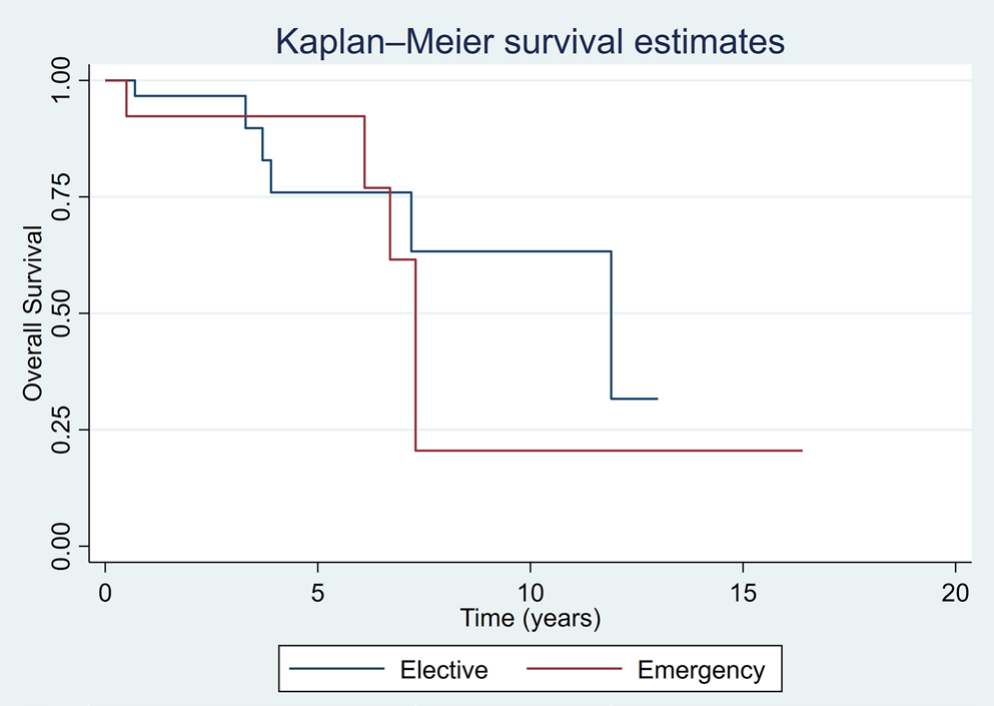
Overall survival estimates

**Fig. 4:**
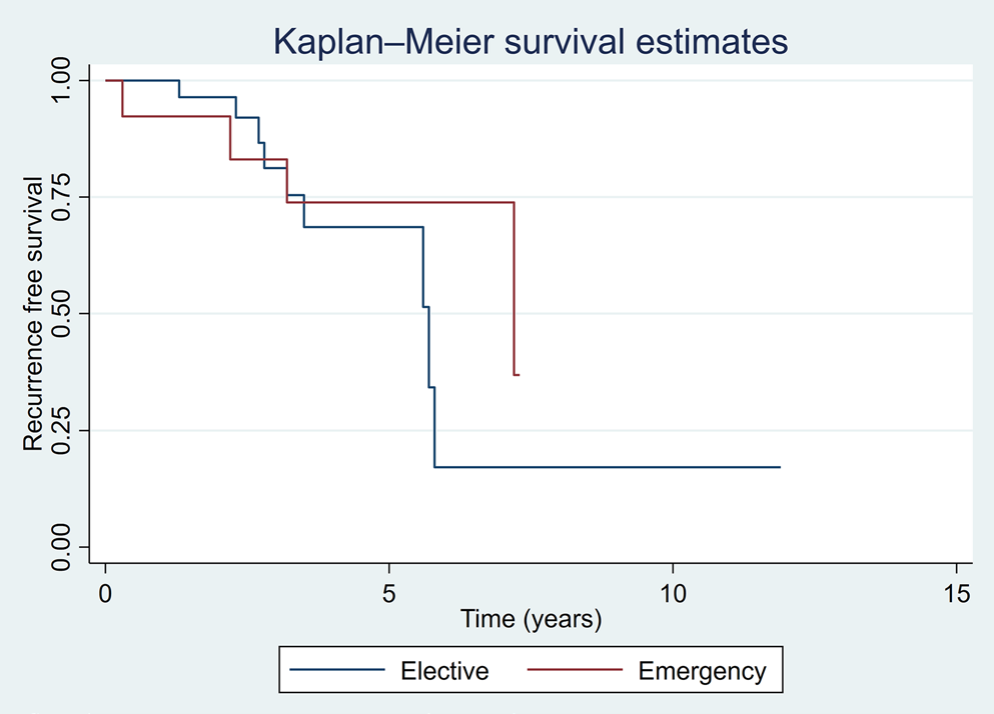
Recurrence free survival

**Fig. 5:**
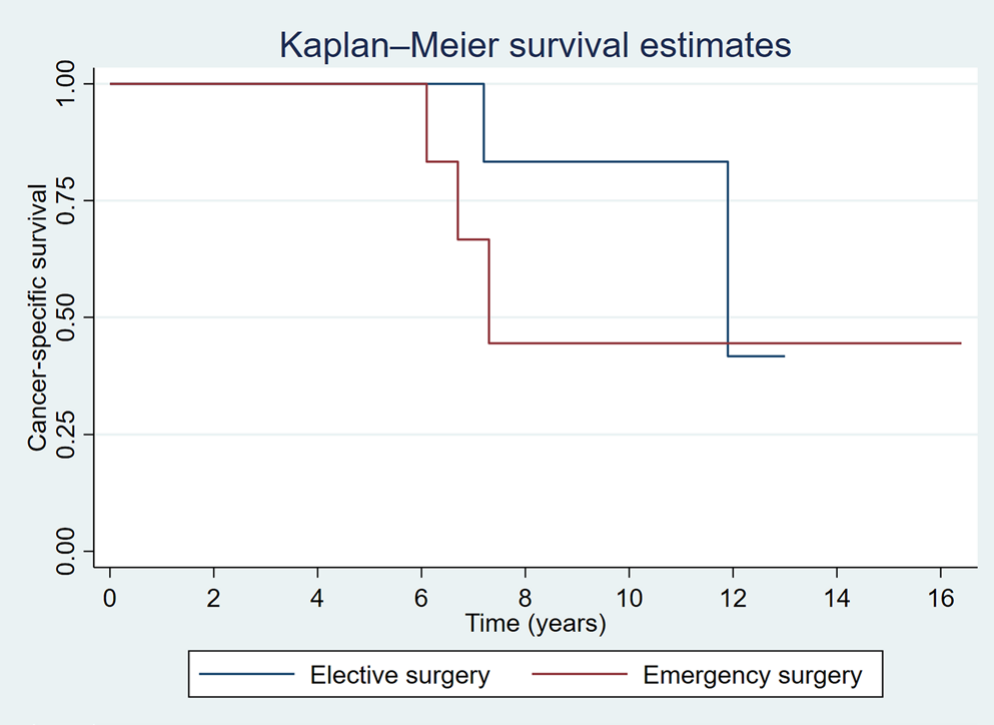
Cancer-specific survival

Recurrence rate following elective and emergency surgery during follow-up was comparable between the two groups (29.0% [9/31] vs. 30.8% [4/13]), respectively. Further resection of bowel (completion or for recurrence) was more common after emergency surgery (3/13 [23.1%] vs. 2/31 [6.5%], *p* = 0.144). Overall proportions suggest higher disease-specific mortality following an emergency resection, but it was statistically insignificant. Mortality was analysed by cause- siNET-related deaths: elective 2/31 (7%) vs. emergency 3/13 (23%) and other-causes related deaths: elective 4/31 (13%) vs. emergency 2/13 (15%). Kaplan-Meier curve showed no association between overall survival, recurrence-free survival and cancer-specific survival and intervention timing as shown in Figs. 3, 4 and 5.

**Table 2:** Oncological outcomes

Group	Elective	Emergency	*P*-value*
Primary outcomes			
Overall survival, years			0.621
- 75% survival time	7.2	6.7	
- Median survival time	11.9	7.3	
Recurrence free survival, years			0.335
- 75% survival time	3.5	3.2	
- Median survival time	5.7	7.2	
Cancer-specific survival, years			0.355
- 75% survival time	11.9	6.7	
- Median survival time	11.9	7.3	
Secondary Outcomes			
Resection staging (n | %)			0.140
- Complete (R0)	14 | 45%	6 | 46%	
- Residual microscopic disease (R1)	10 | 32%	1 | 8%	
- Residual macroscopic disease (R2)	7 | 23%	6 | 46%	
Adjuvant systemic therapy (n | %)			0.247
- None	18 | 58%	4 | 31%	
- Adjuvant	5 | 16%	3 | 23%	
- After disease progression	8 | 26%	6 | 46%	
Small bowel obstruction (n | %)			0.619
- Malignant small bowel obstruction	0	0	
- Non-malignant small bowel obstruction	4 | 13%	1 | 8%	
Bowel ischemia (n | %)	0	0	
Carcinoid (n | %)			0.113
- No	21 | 68%	7 | 54%	
- Post-operatively	6 | 19%	6 | 46%	
- Post disease progression	4 | 13%	0	
Follow up time, years (mean ± sd)	4.1 ± 3.3	5.7 ± 3.9	0.163

*Log-rank test for equality of survivor function

### Systemic Therapy

The distribution of systemic therapy did not differ significantly between emergency and elective resections as shown in Table 2. Systemic therapy association with R-status and mortality is summarised in Table 3. Patients with R2 disease were significantly more likely to receive adjuvant or progression-directed systemic therapy. Nonetheless, there was no significant association observed between patients received systemic therapy and mortality.

**Table 3:** Systemic therapy association with R-status and mortality

Systemic therapy	*R*-status			Mortality		
	R0 (20/44)	R1 (11/44)	R2 (13/44)	Alive (33/44)	End-stage siNET death (5/44)	Other-cause death (6/44)
Nil (22/44)	75%	45.5%	15.4%	77.3%	4.5%	18.2%
Adjuvant therapy (8/44)	0%	9.1%	53.8%	75%	12.5%	12.5%
Post-recurrence/progression (14/44)	25%	45.5%	30.8%	71.%	21.4%	7.1%

### Clinical Outcomes

Post operative carcinoid syndrome status was categorised into resolved, persistent and new after disease progression. Separate analysis of post operative carcinoid syndrome outcome states showed no statistical significance across the two groups. All recorded small bowel obstruction events were non-malignant, and incidence were similar between the two groups. No malignant bowel obstruction or bowel ischemia events were recorded in either group. 

### Primary Oncological Outcomes Prognostic Factors

Prognostic factors were analysed but was limited by the small sample size. Only advanced age (> 70 years) and recurrence were found to be predicters of mortality (Table 4). The timing of surgery was not found to be a significant predictor of mortality but this may be due to the small sample size. 

**Table 4:** Association between selected predictors and mortality

Predictor	Hazard Ratio	*P*-value*
Age over 70 years	*8.93*	*0.041*
Sex, male	1.12	0.898
Emergency surgery	4.73	0.141
Adjuvant systemic therapy	1.02	0.970
Recurrence	*0.04*	*0.019*
Tumour grade	2.98	0.205
Macroscopic residual disease	0.23	0.300

*Cox proportional hazards regression model, italic denotes significant association with *P*-value < 0.05. This model shows a good fit to the data with Prob > chi2 = 0.0296

## Discussion

In this study elective and emergency surgeries demonstrated comparable outcomes in terms of survival, recurrence and overall mortality. Although there was a trend toward higher R2 resection rates and increased disease-specific mortality following emergency surgery, these differences did not reach statistical significance. The type of surgery (elective vs. emergency) did not influence the use of systemic therapy. Instead, residual disease status (R status) was the primary determinant with R2 patients being more likely to receive adjuvant or progression-directed treatment. Systemic therapy did not significantly impact overall survival or disease-specific mortality, although patients receiving therapy generally had more advanced disease at baseline. Postoperative outcomes and bowel complication rates were similar between elective and emergency resections. Carcinoid syndrome outcomes also did not differ significantly by surgical setting. All postoperative bowel obstruction events were benign, with no malignant or ischemic complications observed.

Our institution follows the international guidelines in the management of siNET with all patients diagnosed with a diagnosis of siNET discussed at the gastrointestinal multidisciplinary meeting. All patients with a resectable and localised siNET would be offered an elective resection if fit for surgery. Patients with metastatic disease will also be offered surgery of the primary and metastatic disease if possible to reduce the risk of acute complications to prevent the need for emergency surgery. The use of adjuvant therapy is based on the residual disease. Patients are followed regularly as per guidelines. Patients undergoing emergency surgery are those that present with an acute presentation in those opting for initial medical management or more commonly with the first presentation of siNET as an emergency presentation. These are often not known at the time of surgery and may account for the higher risk of R2 resection in this group.

Given the nature of acute presentation in the emergency group, preoperative functional imaging was not feasible. This limited work-up likely contributed to the higher proportion of R2 resections in the emergency group, though not statistically significant. However, several studies have suggested that incomplete cytoreduction as in R2 resection, is associated with poorer long-term outcomes in siNETs [2].

Although elective resection is associated with more complete staging and cytoreductive intent (including nodal and liver disease), there was no significant difference in carcinoid symptoms resolution between the two groups. This likely reflects that carcinoid symptom control in siNETs also depends on medical suppression of tumour hormone secretion, rather than on cytoreduction only [13]. Finally, the absence of post-operative malignant small bowel obstruction and bowel ischemia in our cohort underscores the role of resection in alleviating and preventing local disease-related complications.

There are conflicting reports on the effect of emergency surgery on oncological outcomes in patients with siNET. Some studies demonstrated no difference compared to elective surgery, while others reported worse outcomes. Manguso et al. found lower lymph node resection rates in emergency resection compared to elective resections, but no significant difference in recurrence rate, disease free survival and overall survival [14]. Snorradottir et al. found that emergency surgery was associated with a six-fold increase in the risk of death within the first 12 months post-surgery due to more severe complications in the emergency resection [15]. However, no long-term mortality difference was observed after this period [15]. Le Roux et al. found that emergency surgery was linked to a fourfold increased risk of relapse compared to elective surgery [16]. ​Similarly, Butz et al. showed a 5-year overall survival (OS) was lower in the emergency group (85.2% vs. 89.5%; *p* = 0.023) and 5-year progression-free survival (PFS) was also markedly lower (26.7% vs. 52.5%; *p* = 0.018) [17]. In multivariable regression analysis, emergency surgery was negatively associated with OS [17].

Our study showed no significant difference in complication between the elective and emergency surgical groups. There were no operative or in-hospital mortality in either group. This is in contrast to the study by Snorradottir et al. who found more severe complications in the emergency surgery group [15].

There are limitations to this study including the small cohort in a single centre that limits statistical power and generalisability as well as increases the risk of type II error. Despite this limitation, the strength of this study is a consistent and standardised management of patients with SiNET with a proportionally small number of patients with incomplete data. These factors enhance internal validity and reduce heterogeneity. In addition, the retrospective nature of the study introduces documentation biases, particularly in symptom reporting and cause-of-death attribution. Despite these limitations this study provides additional data for the management of these uncommon cancers. Elective and planned management of these patients after a complete staging investigation in a higher volume centre by expert surgeons remains a standard of care when a diagnosis of siNET is made. Multicentre prospective projects to validate our findings in larger and more diverse populations may be difficult due to the uncommon nature of these cancers. This may be overcome by interrogating registry data to answer this important question.

## Conclusion

This is one of a few siNET specific studies that analyse the outcomes of emergency and elective resections, offering valuable insight into a rare but clinically significant scenario. Although emergency surgery was associated with limited preoperative staging and a higher proportion of R2 resections, no statistically significant differences in overall survival, recurrence-free survival, carcinoid symptom resolution or local complications were observed in our small cohort. However, these results need to be interpreted cautiously due to the small sample size that may be underpowered to detect significant differences. Nevertheless, the observed trends toward higher disease-specific mortality and incomplete cytoreduction in the emergency cohort underscore the importance of timely diagnosis, comprehensive preoperative workup and planned elective surgery whenever feasible.

## Data Availability

The data generated and analysed in this study can be obtained from the corresponding author upon reasonable request.
